# A multi-docking strategy for robotic LAR and deep pelvic surgery with the Hugo RAS system: experience from a tertiary referral center

**DOI:** 10.1007/s00384-024-04728-2

**Published:** 2024-10-01

**Authors:** Matteo Rottoli, Tommaso Violante, Giacomo Calini, Stefano Cardelli, Marco Novelli, Gilberto Poggioli

**Affiliations:** 1 Surgery of the Alimentary Tract, IRCCS Azienda Ospedaliero-Universitaria Di Bologna, Bologna, Italy; 2 Department of Medical and Surgical Sciences, Alma Mater Studiorum - University of Bologna, Via Massarenti 9, 40138 Bologna, Italy; 3 School of General Surgery, Alma Mater Studiorum- University of Bologna, Bologna, Italy; 4 Division of Colon and Rectal Surgery, Mayo Clinic, Rochester, MN USA; 5 Department of Statistics, Alma Mater Studiorum - University of Bologna, Bologna, Italy

**Keywords:** Robotic surgery, Low anterior resection, Rectal cancer, Medtronic Hugo RAS, Multi-docking strategy, Learning curve

## Abstract

**Introduction:**

In June 2023, our institution adopted the Medtronic Hugo RAS system for colorectal procedures. This system’s independent robotic arms enable personalized docking configurations. This study presents our refined multi-docking strategy for robotic low anterior resection (LAR) and deep pelvic procedures, designed to maximize the Hugo RAS system’s potential in rectal surgery, and evaluates the associated learning curve.

**Methods:**

This retrospective analysis included 31 robotic LAR procedures performed with the Hugo RAS system using our novel multi-docking strategy. Docking times were the primary outcome. The Mann–Kendall test, Spearman’s correlation, and cumulative sum (CUSUM) analysis were used to assess the learning curve and efficiency gains associated with the strategy.

**Results:**

Docking times showed a significant negative trend (*p* < 0.01), indicating improved efficiency with experience. CUSUM analysis confirmed a distinct learning curve, with proficiency achieved around the 15th procedure. The median docking time was 6 min, comparable to other robotic platforms after proficiency.

**Conclusion:**

This study demonstrates the feasibility and effectiveness of a multi-docking strategy in robotic LAR using the Hugo RAS system. Our personalized approach, capitalizing on the system’s unique features, resulted in efficient docking times and streamlined surgical workflow. This approach may be particularly beneficial for surgeons transitioning from laparoscopic to robotic surgery, facilitating a smoother adoption of the new technology. Further research is needed to validate the generalizability of these findings across different surgical settings and experience levels.

**Supplementary information:**

The online version contains supplementary material available at 10.1007/s00384-024-04728-2.

## Introduction

Despite advancements in chemotherapy and radiotherapy, low anterior resection (LAR) remains the cornerstone treatment for rectal cancer [1–4]. This procedure, pioneered by Mayo and Dixon and popularized by Heald with the introduction of total mesorectal excision (TME) [5], has undergone numerous technical refinements over the years.

One major advancement was the introduction of laparoscopic LAR, which demonstrated superior short-term outcomes compared to open surgery, including reduced blood loss, shorter hospital stay, fewer wound complication, and faster recovery of bowel function [6–10].Long-term benefits, such as disease-free and overall survival, have also been observed, establishing laparoscopic LAR as the gold standard [3].

Robotic surgery represented the next evolution of laparoscopic LAR, rapidly gaining popularity due to its enhanced 3D visualization, ergonomics, and precision in the pelvic region [11–14].

The Medtronic Hugo™ Robotic-Assisted Surgery (RAS) (Medtronic, Minneapolis, MN, USA) system is a novel robotic platform which has been recently certified for general surgery. The novel characteristics of the platform include an open console and a modular design, which could offer potential advantages for multi-quadrant abdominal procedures. Furthermore, the system’s four independent robotic arms empower surgeons to tailor docking configurations to their preferences [15,16]. On the other hand, such flexibility might pose a challenge in terms of finding the right set-up for complex procedures, such as LAR, which require different steps on multiple abdominal quadrants.

In June 2023, the Medtronic Hugo RAS system was implemented in the colorectal activity in our Institution. This article aims to share our personalized and clinically validated multi-docking strategy for robotic LAR and deep pelvic procedures, leveraging the Hugo RAS system’s independent robotic arms maximizing its use in rectal procedures.

## Methods

### Study design

This study is a retrospective analysis of prospectively collected data on robotic LAR procedures completed using the Hugo RAS system at our institution (Sant’Orsola Hospital, IRCCS Azienda Ospedaliero-Universitaria di Bologna, Bologna, Italy) from September 2023 to June 2024. The procedures were carried out by a single surgeon experienced in laparoscopic colorectal procedures (> 800 procedures), robotically naïve. Docking was carried out by the first surgeon and the assistant surgeon in parallel from both sides of the patient. The study was designed and reported according to the STROBE guidelines (supplementary materials).

### Data collection

The primary data collected focused on docking times for each phase of the multi-docking strategy. We also included clinical data, encompassing patient characteristics (sex, age, body mass index (BMI), underlying disease, ASA score), operative variables (procedure type, operative time, number of conversions, intraoperative complications, number of high priority alarms (RED)) and postoperative variables (length of stay, 30-day morbidity, 30-day readmission, 30-day reoperations).

### Rationale and development of the multi-docking strategy

The Hugo RAS platform, while equipped with a detailed user guide and several Medtronic-tested docking configurations for various abdominal procedures, presented challenges in our real-world surgical experience. Many of these preset configurations did not seamlessly translate to actual operations, often requiring surgeons to adopt unfamiliar approaches or make last-time adjustments to achieve desired outcomes while avoiding external or internal collision. This could potentially diminish the comfort and intuitive experience that robotic platforms are designed for.

Additionally, in procedures like LAR, the necessity to mobilize the splenic flexure for a tension-free anastomosis poses a unique challenge for robotic surgery, which is typically optimized for smaller surgical fields.

To address these limitations and to optimize instrument reach, minimize arm collisions, and ensure a seamless surgical workflow, we developed a personalized multi-docking approach for LAR and pelvic surgery using what we called a W-I-shaped port placement. This approach is based on the experience developed in laparoscopic surgery and aims to reproduce a similar triangulation of the instruments and obtain a similar view of the anatomy in the different surgical fields. Therefore, the robotic setup involves three distinct docking configurations:

1. First docking: splenic flexure mobilization
2. Second docking: vascular control
3. Third docking: LAR with TME

Each docking configuration is tailored to the specific surgical goals of that phase, enhancing efficiency and precision.

### The W-I-shaped ports placement

After induction of pneumoperitoneum with a Veress needle at Palmer’s point, the abdomen is insufflated to a pressure of 10–12 mmHg. Once the insufflation is completed, an 11 mm trocar for the camera is placed using the visual access technique just above the umbilicus (trocar 1). On the same vertical axis, an 8 mm robotic trocar is placed in the epigastrium (trocar 2) and a 12 mm Airseal (CONMED Corporation, Utica, New York) trocar is placed in the suprapubic area (trocar 3), where a Pfannenstiel incision will be performed later in the procedure. These three trocars form the central “I” shape on the abdomen.

**Fig. 1:**
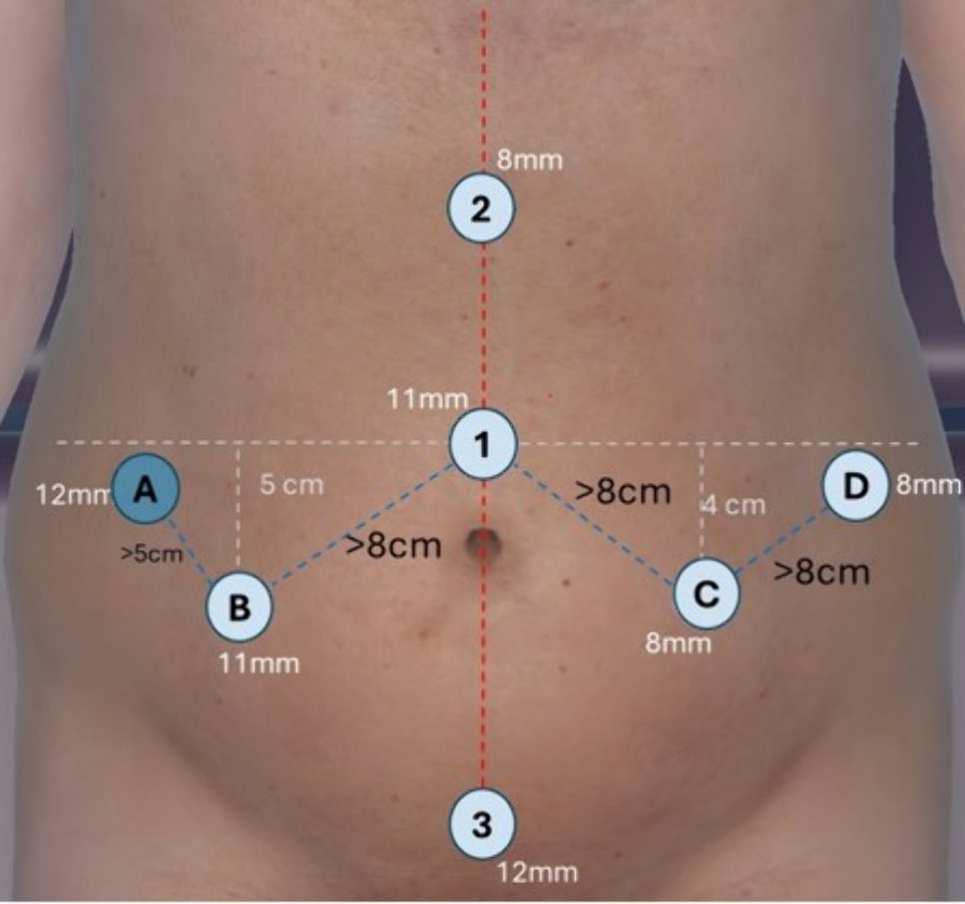
Trocar placement

Four additional trocars are placed in a W-shaped pattern to complete the port placement (Fig. 1). On the right hemiabdomen, an 11 mm robotic trocar (trocar B) is placed 5 cm vertically below the supraumbilical port and at least 8 cm diagonally in the right iliac region. Laterally and superior to this port, a 12-mm assistant port (trocar A) is placed at least 5 cm away in the right lumbar region. On the left hemiabdomen, an 8 mm robotic port (trocar C) is placed in the left iliac region 4 cm horizontally and at least 8 cm diagonally from trocar 1. Laterally and superior to this port, at least 8 cm away, another 8 mm robotic port (trocar D) is placed in the left lumbar region.

Prior to docking the robotic system, a thorough laparoscopic exploration of the abdomen is performed, with lysis of adhesions if needed. The omentum and small bowel are then carefully mobilized towards the right side of the peritoneal cavity as necessary.

#### First docking: the splenic flexure mobilization

**Fig. 2:**
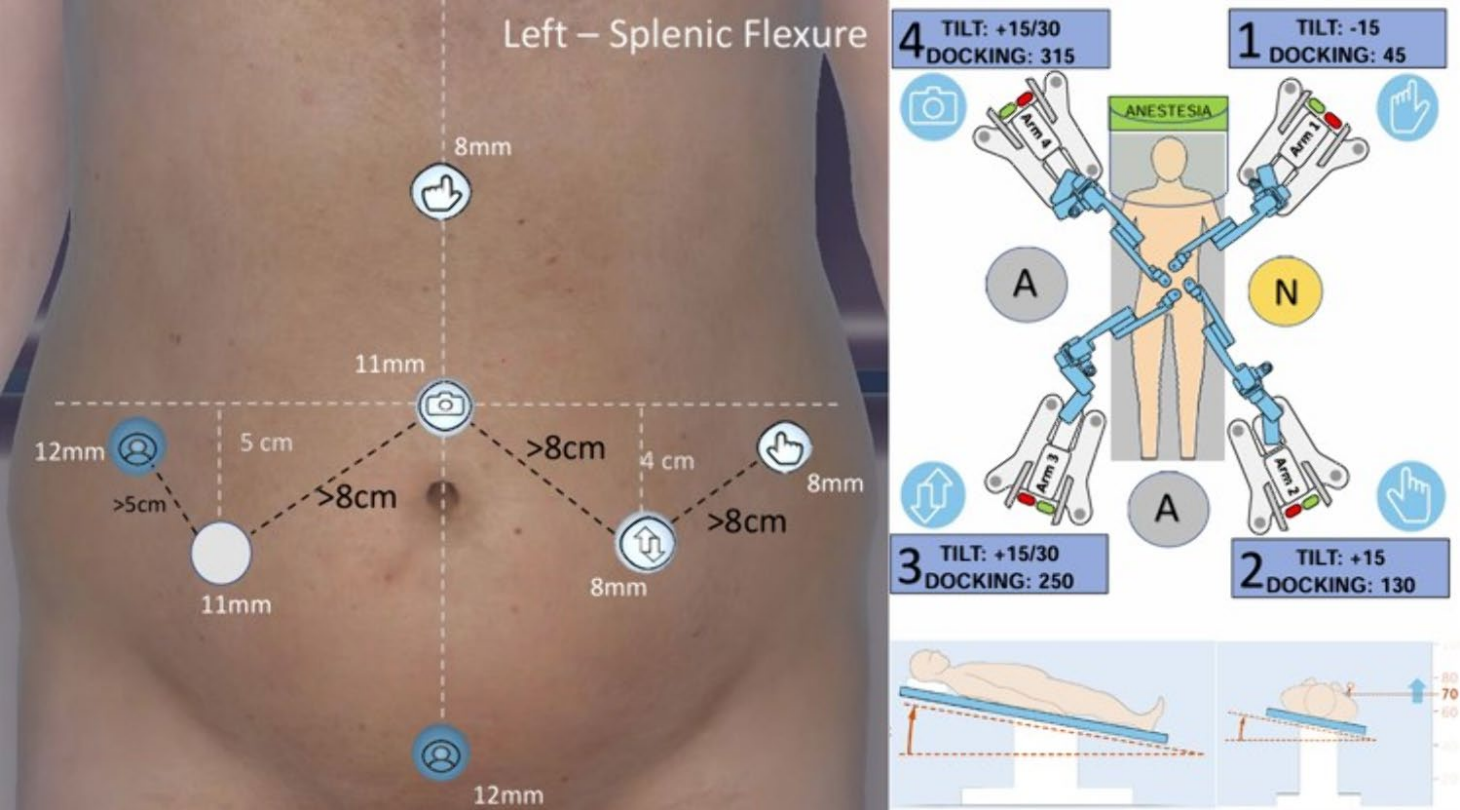
Docking setup for splenic flexure mobilization

The patient is positioned in a 10° anti-Trendelenburg position with a 10° right tilt (Fig. 2). Throughout all docking stages, the operating table height is set above the 70 marks on the robotic cart to ensure the lowest trocar incision is positioned. This adjustment, as indicated by Medtronic, guarantees arms the appropriate tilt and the largest range of motion available.

Arm 1 is docked in trocar 2 (epigastric) with a tilt angle of − 15° and a docking angle of 45°. Arm 2 is docked in trocar D (right lumbar) with a tilt angle of + 15° and a docking angle of 130°. Arm 3 is docked in trocar C (left iliac region) with a tilt angle of + 15/ + 30° (adjusted based on patient build) and a docking angle of 250°. Arm 4 uses the same tilt angle as arm 3 and is docked in trocar 1 (supraumbilical) with a docking angle of 315°. Once the arms are securely docked and the confirmation is given to the system, the instruments are assigned as follows in a double right-hand configuration:

- *Arm 1*: Bipolar forceps
- *Arm 2*: Monopolar curved shears
- *Arm 3*: Cadiere forceps
- *Arm 4*: 30° camera

After completing the docking in this phase, the surgeon utilizes this configuration to mobilize the splenic flexure and to divide the gastrocolic ligament. This ensures adequate mobilization of the distal transverse and the left colon, guaranteeing easy access to the large bowel within the pelvis for the subsequent colorectal or coloanal anastomosis. During this phase, if required, the assistant can facilitate the dissection by applying further traction to the colon through trocar A, B, or 3 (Fig. 1). This aids the surgeon in achieving a smooth and efficient mobilization of the splenic flexure, which is usually taken down from lateral to medial. A proper triangulation is obtained and the surgeon controls two instruments (monopolar shears and Cadiere forceps) on their right hand, allowing a proper traction and counter-traction on the bowel.

#### Second docking: vascular control

**Fig. 3:**
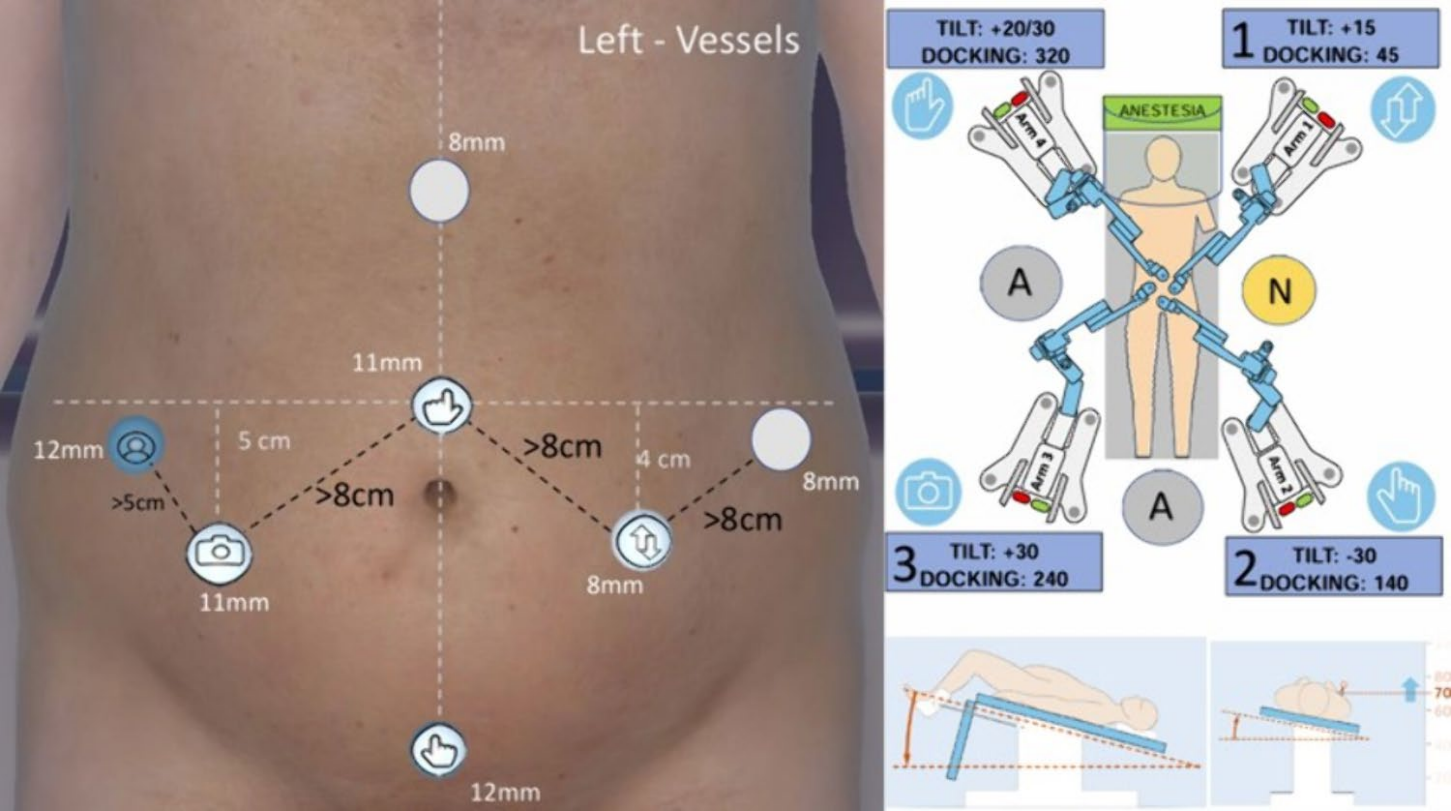
Docking setup for vascular control

After completing the splenic flexure mobilization, all robotic arms are undocked. The robotic carts remain in the same position, optimizing the redocking time. The patient is positioned in a steep Trendelenburg position maintaining the 10° of right tilt, and the table height is adjusted to ensure the lowest trocar incision remains above the 70 mark on the robotic arms (Fig. 3).

The docking configuration is then modified as follows:

- *Arm 1*: Tilt angle changed to + 15°, redocked into trocar C (left iliac region) with the same 45° angle.
- *Arm 2*: Tilt angle changed to − 30°, docked into an 8 mm robotic port that is inserted in trocar 3 (the 12 mm suprapubic port) with a 140° angle.
- *Arm 3*: Tilt angle changed to + 30° (if not already set), docked into trocar B (right iliac region) with a 240° angle.
- *Arm 4*: Tilt angle changed to + 20/30° (depending on patient build), docked into trocar 1 (supraumbilical) with a 315° angle.

Once the arms are securely docked, the instruments are assigned as follows in a double right-hand configuration:

- *Arm 1*: Cadiere forceps
- *Arm 2*: Monopolar curved shears
- *Arm 3*: 30° camera
- *Arm 4*: Bipolar forceps

In this step of the surgical procedure, the surgeon carries out the vascular control of the inferior mesenteric artery and inferior mesenteric vein (the order of the division is up to the personal preferences of the surgeon), obtaining a lateral view of the aortic plane, similar to the perspective used in laparoscopic surgery (from the right to the left side). The dissection of the left colon is then completed with a medial-to-lateral approach, reaching the plane previously dissected during the mobilization of the splenic flexure. The placement of Hem-o-lok® clips on the vessels, as well as any required suction or further traction, is obtained by the assistant through the trocar A.

It is important to highlight that this docking set-up allows the dissection not only of the left colon, but also of the high-mid rectum, thus carrying out a partial mesorectal excision, without limitations or external conflicts of the instruments. Therefore, in case of a left hemicolectomy or a sigmoid resection, no further redocking will be necessary.

#### Third docking: LAR with TME

**Fig. 4:**
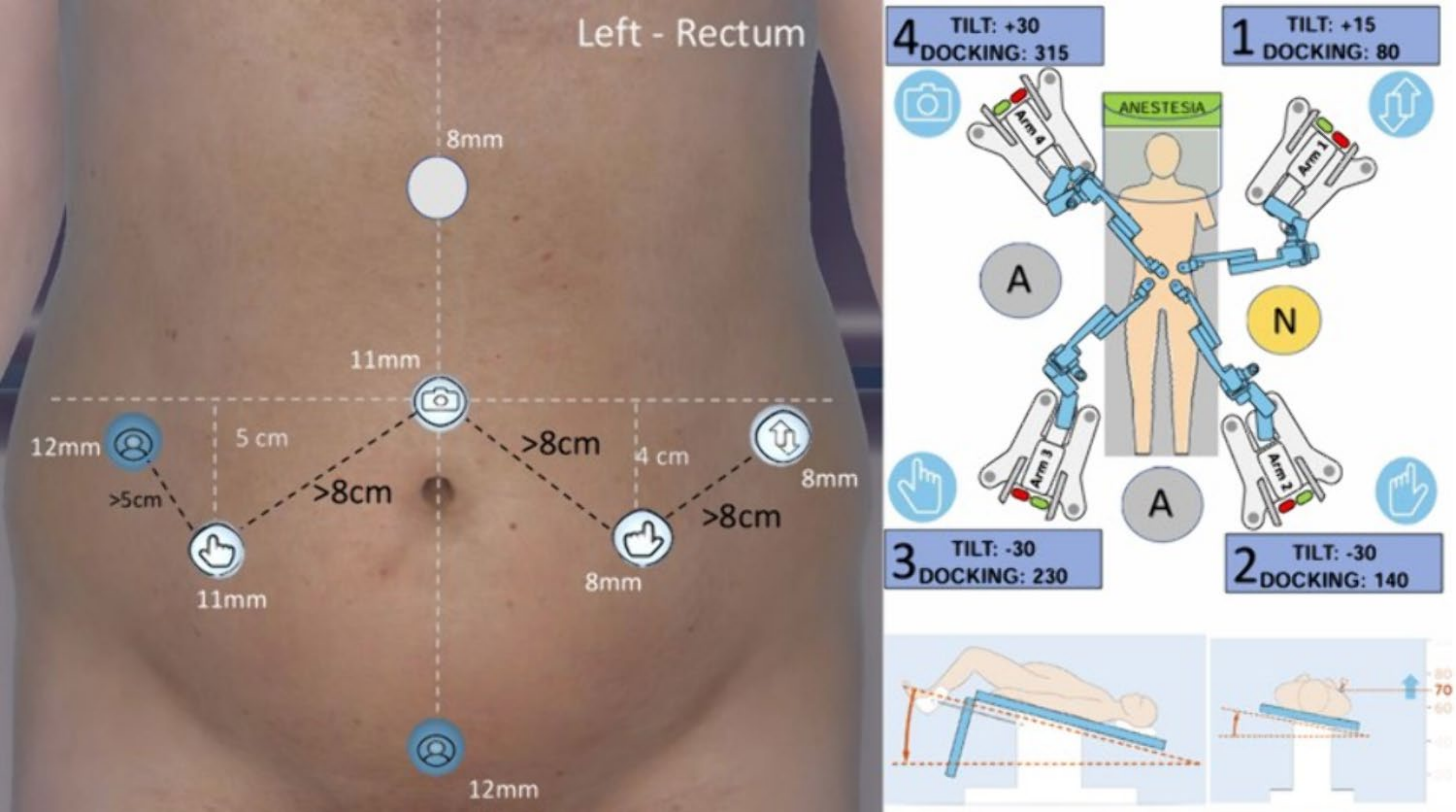
Docking setup for low anterior resection with total mesorectal excision

Once completed the vascular phase the third and last docking is carried out (Fig. 4)as follows:

- *Arm 1*: Tilt angle does not change (+ 15°), redocked into Trocar D (left lumbar) with a docking angle of 80°.
- *Arm 2*:Tilt angle and docking angle do not change (− 15° and 140°, respectively) and it is moved from trocar 3 to trocar C.
- *Arm 3*: Tilt angle changed to − 30°, docked into trocar B (right lumbar) with a 240° angle.
- *Arm 4*: Tilt angle changed to + 30° (if not already set), docked into trocar 1 (supraumbilical) with the same angle as the previous docking (315°).

In this last docking instruments are assigned as follows in a double left-hand configuration:

- *Arm 1*: Cadiere forceps
- *Arm 2*: Bipolar forceps
- *Arm 3*: Monopolar curved shears
- *Arm 4*: 30° camera

This port configuration optimizes instrument reach in pelvis down to the levator ani plane, minimizing collisions and facilitating efficient completion of the LAR with TME. In our practice, the assistant utilizes the trocar 3 (12 mm Airseal suprapubic port) to maintain traction on the rectum during TME using a 10 mm Babcock forceps. The smoke formed during surgery is effectively removed through the Airseal evacuation. Moreover, a fully powered articulated laparoscopic stapler (Medtronic Signia™) could be inserted in the assistant trocars A or 3 to carry out the transection of the rectum according to surgeon preference.

Upon completion of the TME, all robotic arms are undocked. An incision is made at the planned loop ileostomy extraction site, where a retractor (Alexis O Wound retractor, Applied Medical, Rancho Santa Margarita, CA, USA) is placed to facilitate extraction of the colonic stump and which will also serve as the specimen extraction site. The rectum is proximally transected, and the circular stapling anvil is inserted into the colonic stump. The rectum is then returned to the abdomen, the colorectal anastomosis is constructed, and an air-leak test is carried out. An ileostomy is constructed if required by the single case.

We also utilize this robotic port configuration for restorative proctectomy and ileal pouch-anal anastomosis (IPAA) procedures, with some modifications. In this cases, two Alexis retractors are placed as first steps after the end ileostomy in the right iliac fossa and the rectum stump (secured to the suprapubic fascia as for our habits) are taken down [17], and the port configuration is modified by excluding Trocar 2 and the first docking for the splenic flexure mobilization, which is not required.

### Statistical analysis

Descriptive statistics, including mean, median, interquartile range, and standard deviation, were calculated to summarize docking times. To assess the presence of a negative trend in docking times, the non-parametric Mann–Kendall test and Spearman’s rank correlation coefficient were utilized.

Additionally, a cumulative sum (CUSUM) analysis was conducted to monitor for shifts in docking times. The CUSUM analysis was performed twice: first using the mean docking time of all cases as a reference, and then using the mean time of the first 15 cases as a reference. This approach allows for the identification of potential changes in docking times over time, both overall and specifically in the initial phase.

To present clinical variables, categorical variables were expressed as counts and percentage, while continuous variables were summarized using medians and interquartile ranges.

## Results

Thirty-one procedures were recorded using this docking setting. The median docking time for the first operative step was 6 ± 1 min, with a mean of 5.6 ± 1.3 min. A linear regression analysis of the data (Table 1) revealed a negative trend, suggesting that docking time decreased with each subsequent procedure.

**Table 1:**
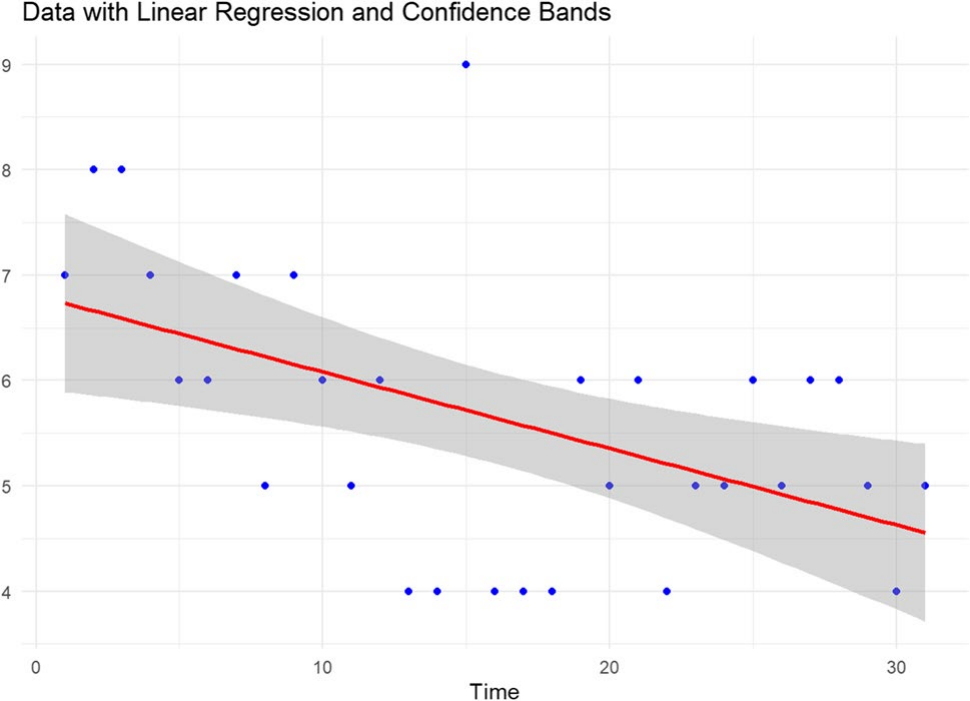
Plot of the docking times with linear regression and confidence bands

To further confirm this negative trend, two non-parametric statistical tests were conducted. The Mann–Kendall test (Tau =  − 0.367, *p* = 0.007) revealed a statistically significant negative trend, while Spearman’s rank correlation test (Rho =  − 0.501, *p* = 0.004) further supported this by demonstrating a statistically significant negative correlation between the order of procedures and docking times.

This learning curve effect was further examined through CUSUM analyses. The first analysis, using the mean docking time of all 31 cases as a reference (5.6 min, Table 2), showed initial docking times above the average, followed by a clear downward trend around the 15th procedure. Given this initial indication of a learning curve, a second CUSUM analysis was conducted using the mean docking time of the first 15 cases (6.3 min, Table 3) as a reference to specifically focus on the initial learning phase. This second analysis confirmed the presence of a distinct shift towards below-average docking times after the initial phase. Both analyses highlight a notable learning curve effect, with operators demonstrating increased proficiency and efficiency over time.

**Table 2:**
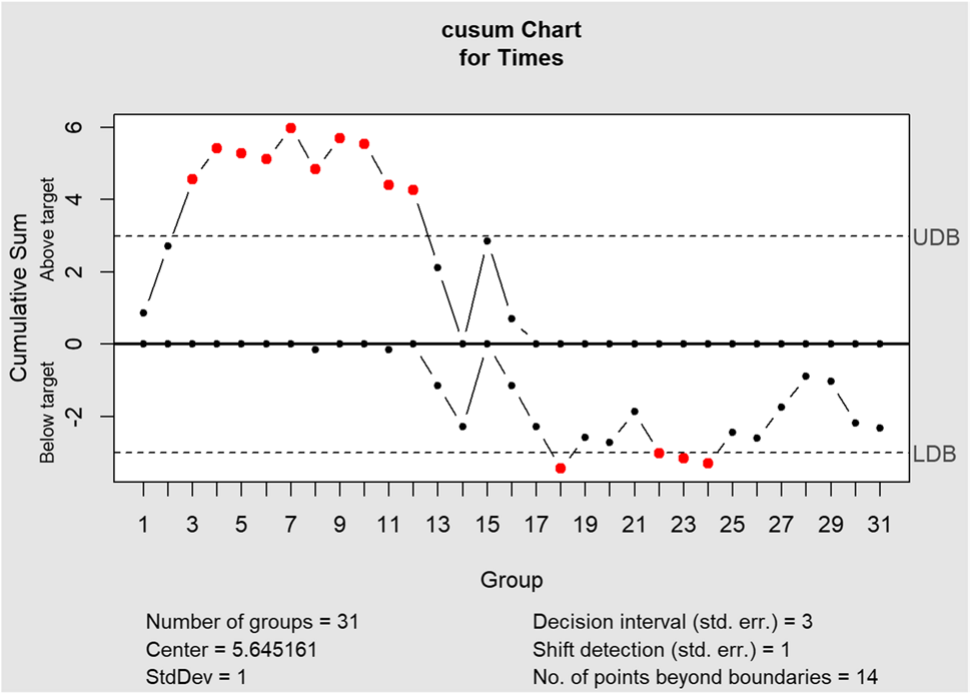
Cumulative sum (CUSUM) analysis referencing the mean of all cases

**Table 3:**
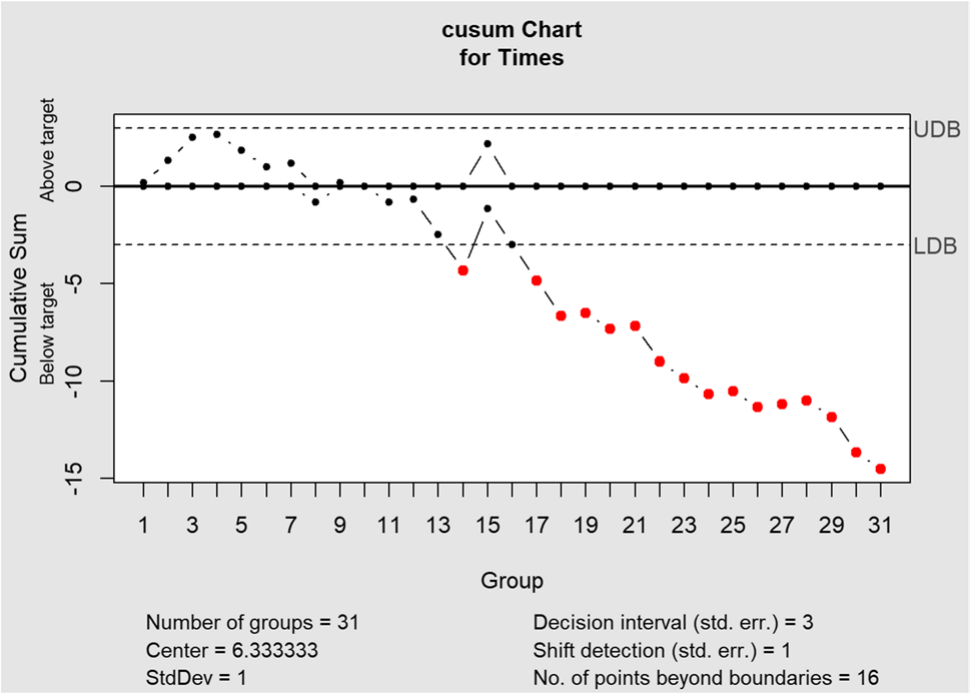
Cumulative sum (CUSUM) analysis referencing the mean of the first 15 cases

### Clinical outcomes

The study consisted of 31 patients with a median age of 52 years (Table 4). The majority were male (*n* = 16) and had a diagnosis of ulcerative colitis (*n* = 21). The median BMI was 21 kg/m^2^, and most patients presented with an ASA score of 2. The most common procedures performed were proctectomies followed by IPAA (*n* = 19) and LARs with partial mesorectal excision (*n* = 6) and total mesorectal excision (*n* = 4).

**Table 4:** Patients’ characteristics, operative variables, and postoperative outcomes

Variables	Patients (*n* = 31)
Male	15 (48.4)
Age (years)	52 (11)
Body mass index (kg/m^2^)	21 (6)
Underlying disease:	
UC	21 (67.7)
Rectal cancer	9 (29.1)
Anal cancer	1 (3.2)
ASA score:	
1	2 (6.5)
2	25 (80.6)
3	4 (12.9)
Neoadjuvant radiotherapy	4 (12.9)
Neoadjuvant chemotherapy	4 (12.9)
Pathological T stage:	
pTx	5 (16.1)
pT1	1 (3.2)
pT2	2 (6.5)
pT3	2 (6.5)
Pathological N stage:	
pN0	9 (29.0)
pN1	1 (3.2)
Tumor location:	
Low	3(9.7)
Medium	1 (3.2)
High	6 (19.4)
Procedures:	
Proctectomy, IPAA, and DLI	19 (61.2)
LAR with PME	6(19.4)
LAR with TME and DLI	4(12.9)
APR	2 (6.5)
Operative time (min)	280 (94)
Conversion	1 (3.2)
Length of stay (days)	7 (2)
30-day morbidity	4 (12.9)
30-day readmission	2 (6.5)
30-day reoperation	1 (3.2)

Categorical variables are number and %; continuous variables are expressed as median and interquartile range

*ASA* American Society of Anesthesiologists, *UC* ulcerative colitis, *IPAA* ileal pouch-anal anastomosis, *DLI* diverting loop-ileostomy, *LAR* low anterior resection, *PME* partial mesorectal excision, *TME* total mesorectal excision, *APR* abdominoperineal resection

The median operative time was 280 min. One conversion to open surgery was necessary due to adhesions. No intraoperative complications or high-priority (red) alarms were encountered. The median length of hospital stay was 7 days.

Postoperative complications within 30 days occurred in four patients. These included one case of ileus requiring nasogastric tube placement, one superficial surgical site infection managed with wound care, and one case of abdominal collection treated with image-guided drainage and antibiotics. Additionally, one patient developed an anastomotic leak due to colonic ischemia, leading to reoperation involving resection of the ischemic colon and creation of an end colostomy.

## Discussion

The independent robotic arms of the Hugo RAS system enabled us to develop a personalized multi-docking strategy that significantly streamlines LAR and IPAA procedures.

Contrary to the perception of many robotic surgeons, our team believes that multi-docking during a procedure, particularly with platforms featuring independent robotic arms, is not a disadvantage but rather a significant opportunity that should be exploited and considered as a standard approach. It grants the platform exceptional flexibility in multi-quadrant abdominal procedures, requiring only minimal adjustments between dockings to optimize machine performance [18]. Our data supports this, with a median docking time of 2.5 min after the initial docking, which always took longer (median ± IQR of 6 ± 1 min). Furthermore, studies in robotic gynecological procedures have demonstrated the benefits of multi-docking, including reduced operative time, blood loss, and improved postoperative recovery, as well as an increased number of harvested lymph nodes [19,20].

Several articles have analyzed the learning curve for docking time, most of them employing the CUSUM analysis. It is interesting to note that previous publications have reported proficiency in docking time achieved after 10 cases for rectal cancer (DaVinci)^21^, 20 cases for prostatectomy (DaVinci) [22], and 17 cases for gynecology (Hugo RAS) [23]. Notably, even with the added complexity of our multi-docking approach, after reaching proficiency, our docking time for the Hugo RAS was comparable to that reported in these studies.

The present setups have been utilized in over 100 colorectal procedures performed by 3 surgeons, all naïve in robotic surgery, without reported major conflicts or need for redocking during the different steps of the procedure [23]. Although a multi-center study should be carried out to confirm the reproducibility of the Bologna setup, the feasibility of the multi-docking approach has been shown in this limited yet significant experience.

This study has several limitations. Firstly, its retrospective nature may introduce biases. Secondly, the study was conducted at a tertiary referral center for rectal cancer and inflammatory bowel disease, which may limit the generalizability of our findings. Finally, the docking setup was tailored to the preferences of our institution’s surgeons, highlighting the need for personalized approaches in robotic surgery.

Hopefully, these results will hopefully encourage more surgical teams to embrace the multi-docking philosophy in robotic colorectal surgery using the Hugo RAS system. This approach is especially useful for the majority of experienced laparoscopic colorectal surgeons who are beginning their robotic experience with this platform. By adopting this method, surgeons can recreate a familiar surgical view, reducing the discomfort associated with readjusting to procedures already mastered in laparoscopy. The next step to explore the different possibilities would be represented by sharing the set-ups with the surgical community [15,16,24–30]. To facilitate this knowledge exchange, the creation of an online, official repository where these dockings can be stored, scored, and commented on by other users of the platform would be invaluable. This collaborative approach could not only push the boundaries of research in the robotic surgical field but also drive the development of optimal docking configurations for each procedure, ultimately improving surgical outcomes and patient care.

## Conclusion

This article offers valuable insights into the potential of multi-docking strategies in robotic surgery, particularly with platforms featuring independent robotic arms, such as the Hugo RAS system. By sharing our docking settings, we aim to foster collaboration within the surgical community, unlocking the full potential of robotic technology and continually improving our collective knowledge of its applications and capabilities.

## Supplementary information

Below is the link to the electronic supplementary material.

Supplementary file1 (PDF 340 KB)

## Data Availability

All data supporting the findings of this study are available within the paper.
